# Popliteal artery pseudoaneurysm of spontaneous occurrence: a case report

**DOI:** 10.1590/1677-5449.202400212

**Published:** 2024-08-23

**Authors:** Wallace Klein Schwengber, Claudia Carolina Schnorr, Guilherme Camargo Winckler, Ricardo Bocchese Paganella, Marco Aurélio Grudtner

**Affiliations:** 1 Universidade Federal do Rio Grande do Sul – UFRGS, Porto Alegre, RS, Brasil.; 2 Hospital de Clínicas de Porto Alegre – HCPA, Porto Alegre, RS, Brasil.

**Keywords:** pseudoaneurysm, popliteal artery, idiopathic, pseudoaneurisma, artéria poplítea, idiopático

## Abstract

Idiopathic popliteal artery pseudoaneurysms are exceedingly rare, posing significant diagnostic challenges due to their elusive etiology. This report presents the case of a 78-year-old female with no history of trauma or orthopedic procedures who was diagnosed with a large pulsatile mass in the right popliteal fossa. Arteriography confirmed a popliteal artery pseudoaneurysm. Despite extensive clinical evaluation, no causative factors were identified, suggesting an idiopathic diagnosis. The patient underwent open surgical repair using a posterior approach, during which the popliteal artery defect was closed using a bovine pericardium patch. Postoperative follow-up revealed proximal patch stenosis, necessitating angioplasty. This case underscores the need for comprehensive diagnostic evaluation of atypical pseudoaneurysm presentations and highlights the complexities involved in managing idiopathic cases, emphasizing the importance of postoperative follow-up to address potential complications.

## Resumo

Pseudoaneurismas idiopáticos da artéria poplítea são extremamente raros, apresentando desafios diagnósticos significativos devido à sua etiologia desconhecida. Este relato apresenta uma paciente de 78 anos, sem histórico de trauma ou procedimentos ortopédicos, diagnosticada com uma grande massa pulsátil na fossa poplítea direita. A arteriografia confirmou um pseudoaneurisma da artéria poplítea. Apesar de uma avaliação clínica extensa, nenhum fator causal foi identificado, levando ao diagnóstico idiopático. A paciente foi submetida a reparo cirúrgico aberto usando uma abordagem posterior, e o defeito da artéria poplítea foi fechado com um remendo de pericárdio bovino. O acompanhamento pós-operatório revelou estenose proximal do remendo, necessitando de angioplastia. Este caso evidencia a necessidade de avaliações diagnósticas abrangentes em apresentações atípicas de pseudoaneurisma e destaca as complexidades do manejo de casos idiopáticos, enfatizando a importância do acompanhamento pós-operatório para abordar complicações potenciais.

## INTRODUCTION

Popliteal artery pseudoaneurysm is a rare clinical entity^1^ in which arterial wall injury usually results from local trauma^2^ or orthopedic procedures^3^,^4^ or is associated with bony deformities, like osteochondromas.^5^ However, idiopathic popliteal artery pseudoaneurysms are exceedingly rare. Some studies suggest a possible link with autoimmune disorders, such as Behçet’s disease, yet occurrences are infrequent and poorly documented.^6^ To date, there are few reports of idiopathic popliteal artery pseudoaneurysms.^6^-^8^

In this report, we present the case of a 78-year-old female with a large idiopathic popliteal artery pseudoaneurysm. Extensive investigation revealed no associated clinical conditions, like Behçet’s disease or other autoimmune disorders. The patient underwent open repair with a bovine pericardium patch. Postoperative follow-up revealed proximal patch stenosis, successfully managed with angioplasty.

The protocol was approved by the Ethics Committee at the Hospital de Clinicas de Porto Alegre (decision number CAAE 71406323.8.0000.5327/GPPG 2023-0231).

## CASE DESCRIPTION

The case involves a 78-year-old brown-skinned female with systemic arterial hypertension, type 2 diabetes mellitus, peripheral artery disease, and a history of ischemic stroke. She reported no history of smoking or alcohol use.

The patient initially presented to the emergency department with symptoms of exercise-related leg pain and paresthesia bilaterally. On physical examination, a small pulsatile mass was noted in the right popliteal fossa, with no palpable distal pulses bilaterally. Additional imaging with color Doppler ultrasound revealed the right popliteal artery measuring 2.0 cm × 1.5 cm, with normal blood flow. During the same hospital admission, the patient was also investigated for a history of 10 kg weight loss (from 64 to 54 kg) over the previous 6 months. Abnormal laboratory findings were C-reactive protein (CPR) of 32 mg/L (reference range of < 5 mg/L) and erythrocyte sedimentation rate (ESR) of 103 mm in the first hour (reference range from 0 to 35 mm). Infection and autoimmune screenings were all normal. Regarding imaging, a CT scan of the thorax and abdomen, mammography, esophagogastroduodenoscopy, and colonoscopy found no cause for her weight loss or elevated inflammatory markers. The patient attributed the weight loss to poor food intake due to dental issues and stress-related family issues. She was discharged with optimized clinical treatment for peripheral artery disease and referred to the Vascular Surgery Service for follow-up.

**Figure 1:**
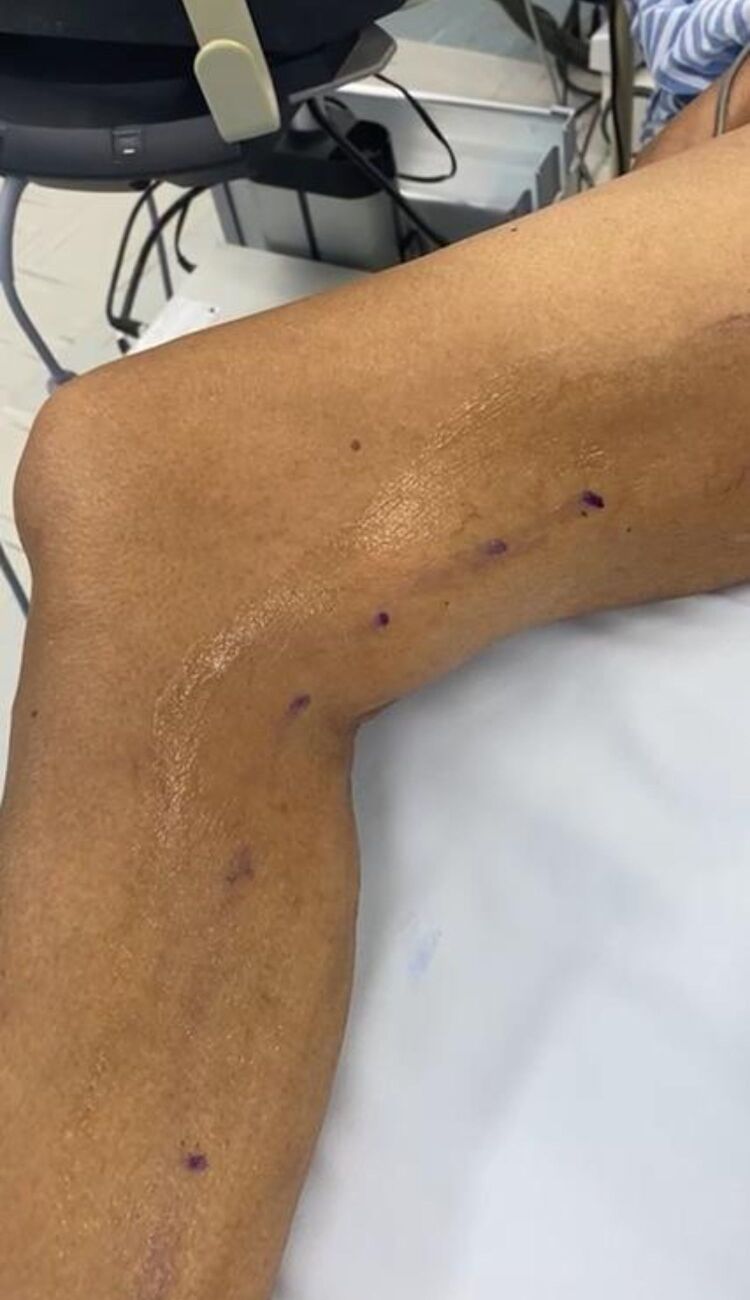
Large pulsatile mass at the right popliteal fossa.

**Figure 2:**
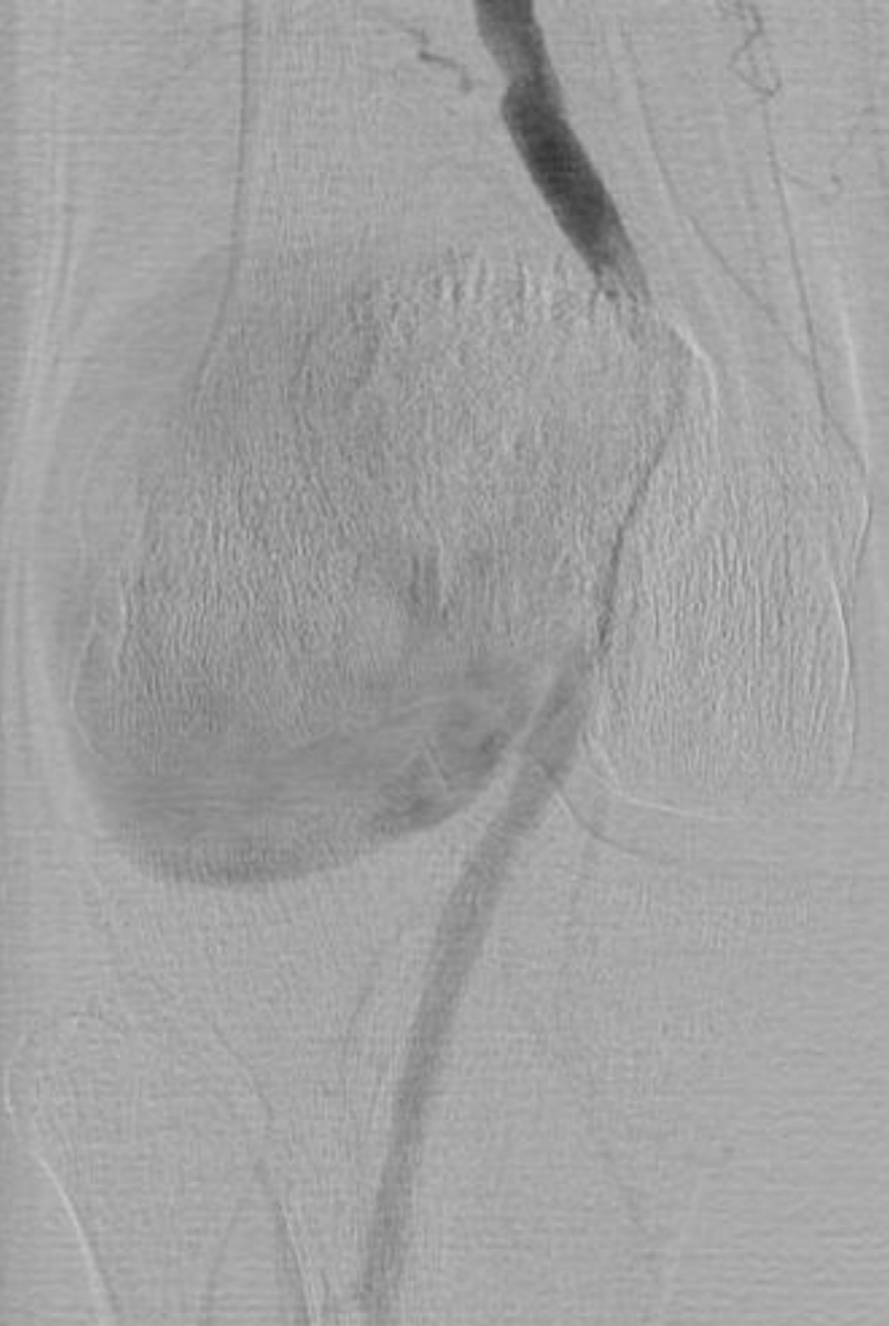
Arteriography of the right leg shows a large pseudoaneurysm of the popliteal artery.

One year later, in a vascular surgery outpatient setting, the patient reported a growing mass at the back of the right knee (Figure 1). On physical examination, there was a large pulsatile mass on the right popliteal fossa. Doppler ultrasound conducted at that time showed a 4 cm dilation of the right popliteal artery. The patient consistently denied any history of trauma, orthopedic surgery, or infection. For detailed assessment, she underwent right leg arteriography, revealing a large pseudoaneurysm in the P2 segment of the popliteal artery (Figure 2).

**Figure 3:**
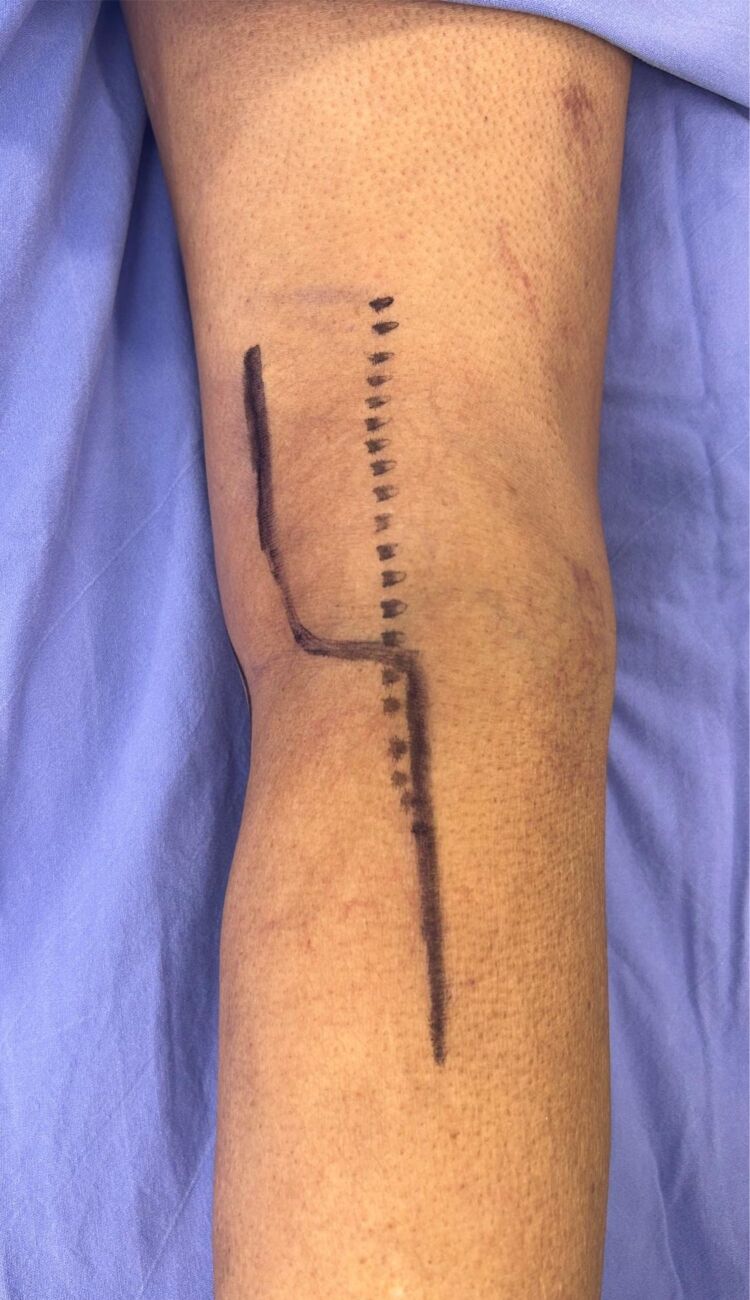
Posterior view of the right leg, showing surgical delimitation before incision.

The patient underwent elective surgery using a posterior approach, chosen for optimal evacuation of the mass. (Figure 3).

**Figure 4:**
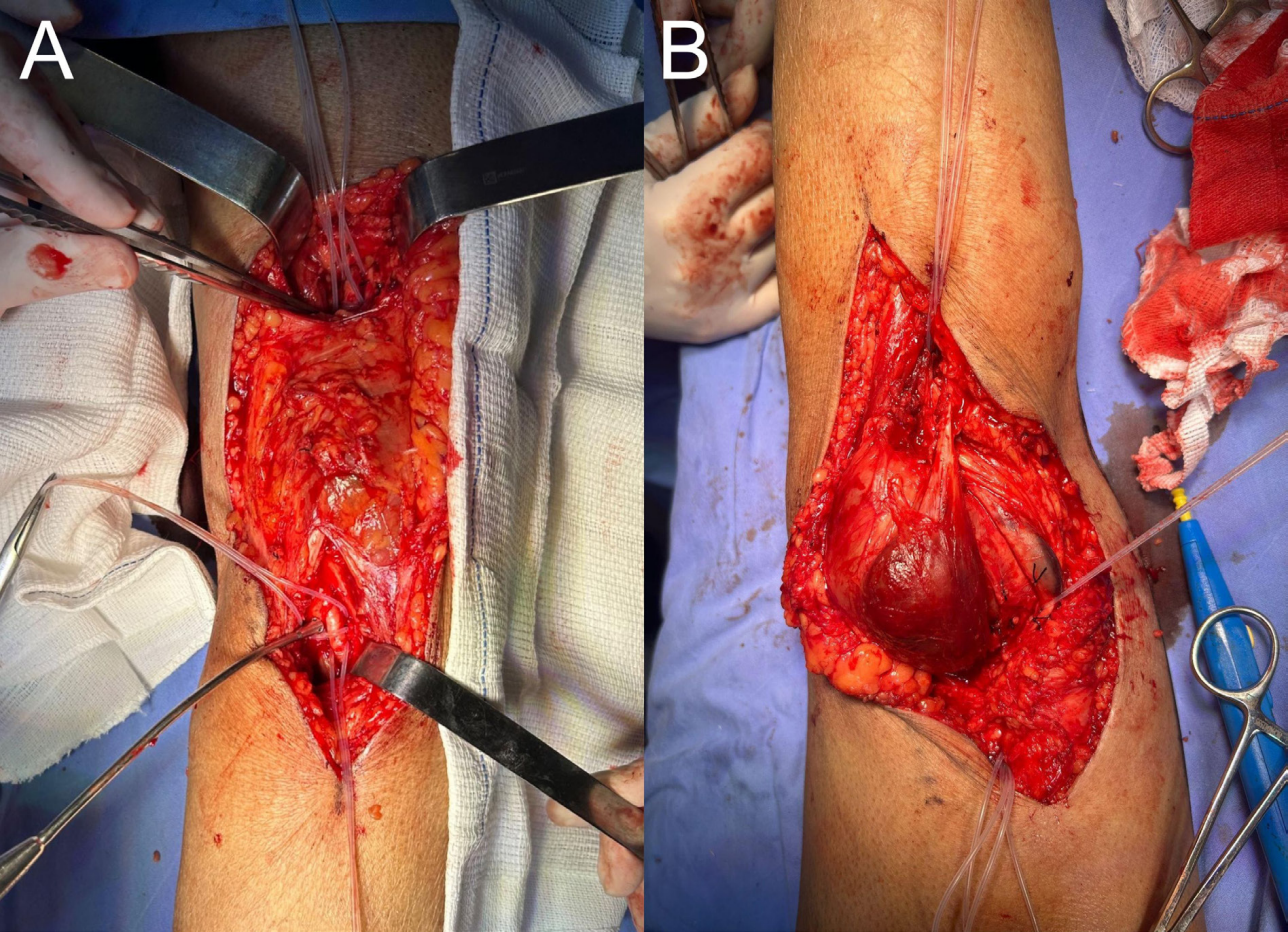
(A) Proximal and distal arterial control; (B) Dissection by planes and exposure of the pseudoaneurysm.

**Figure 5:**
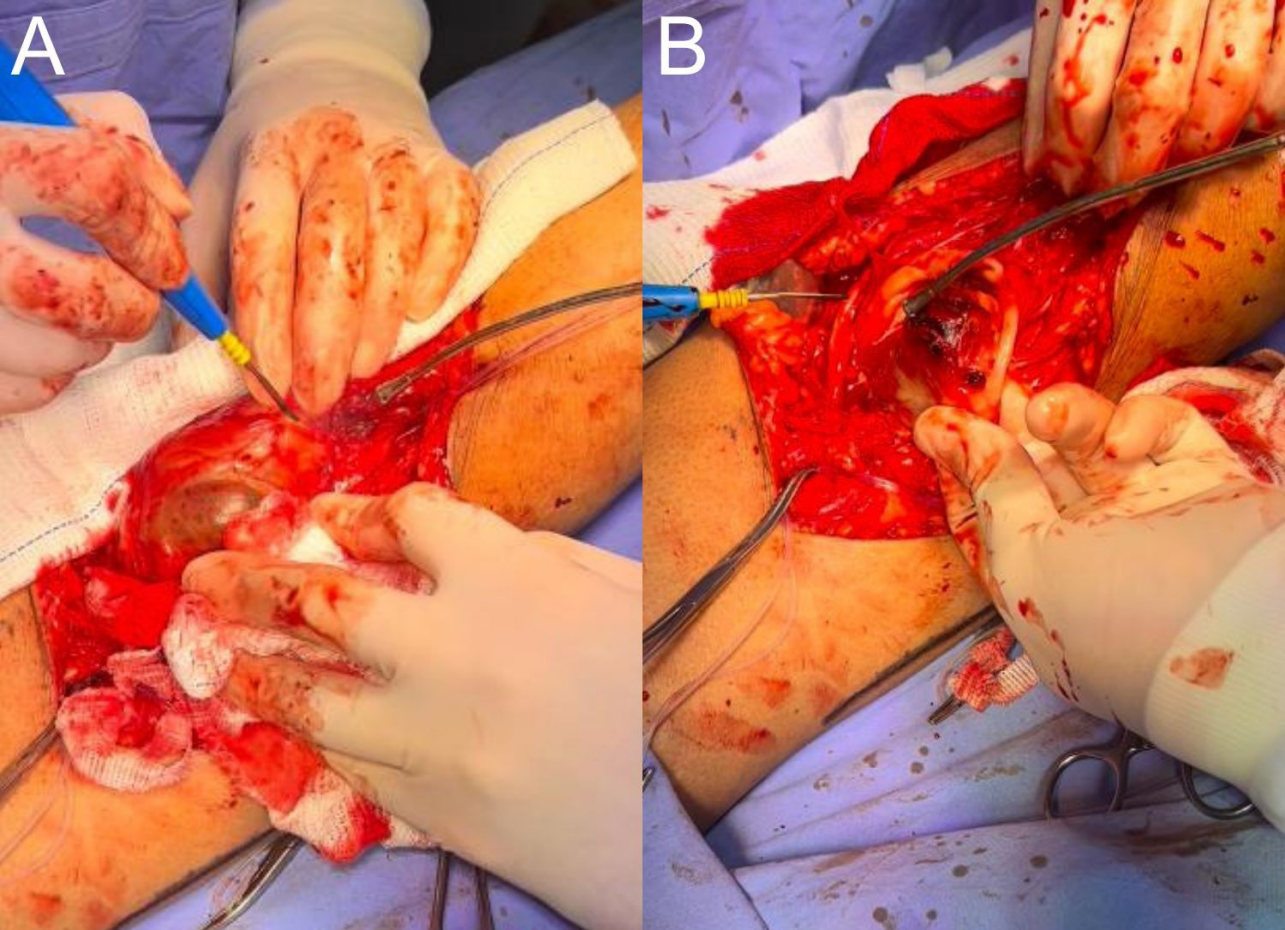
(A) Longitudinal incision of the aneurysm sac; (B) Aneurysm sac without intraluminal thrombi.

**Figure 6:**
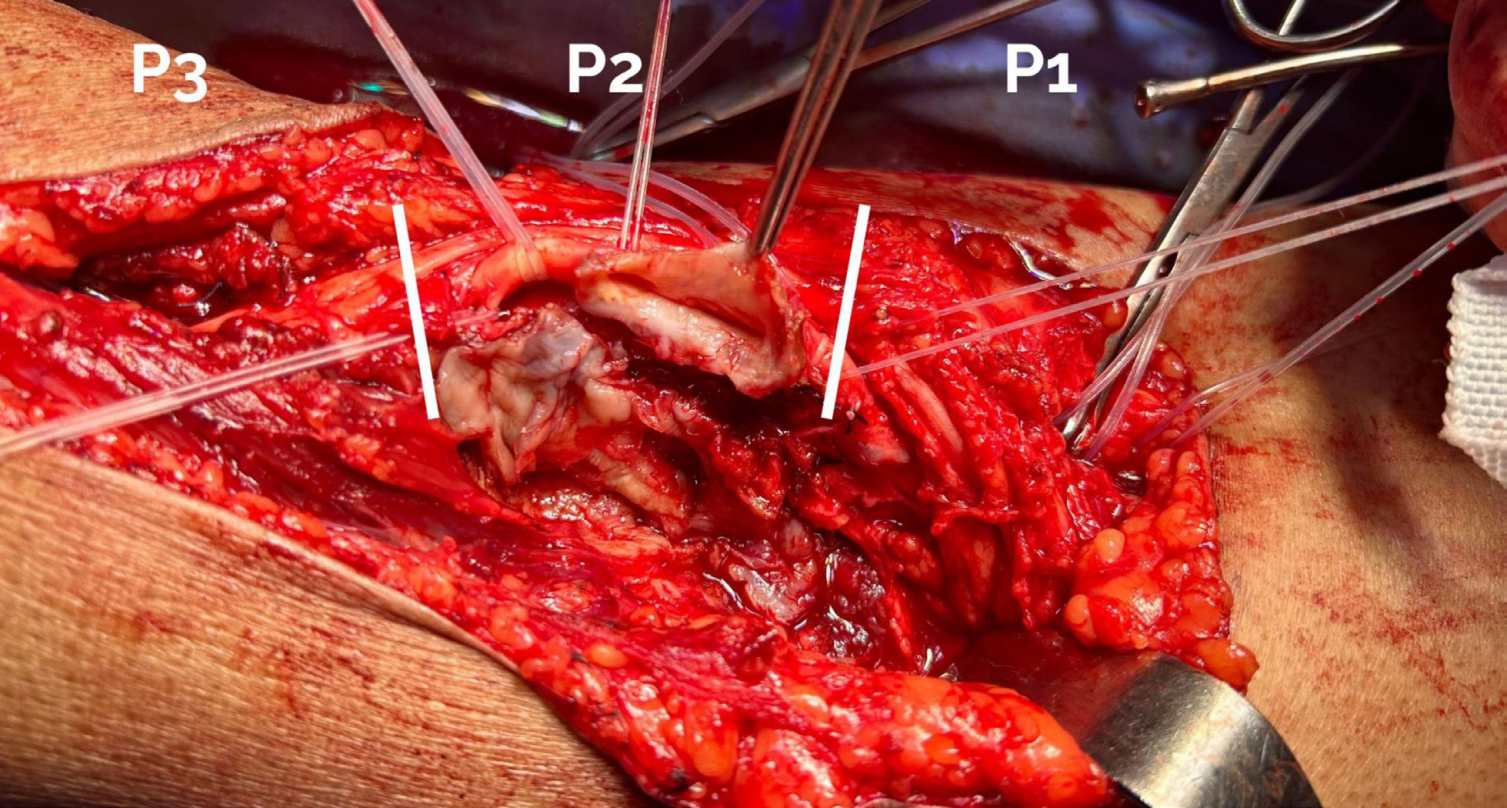
Surgical field after resection of the redundant portion of the aneurysmal sac. P1, P2, and P3 indicate the supracondylar, interline, and infracondylar segments of the popliteal artery, respectively.

Dissection by planes was conducted until the popliteal mass was isolated (Figure 4). After achieving proximal and distal arterial control, a longitudinal incision was made in the aneurysm sac and its contents were emptied (Figure 5). The redundant aneurysmal sac was partially resected until a healthy area of the artery was identified (Figure 6).

**Figure 7:**
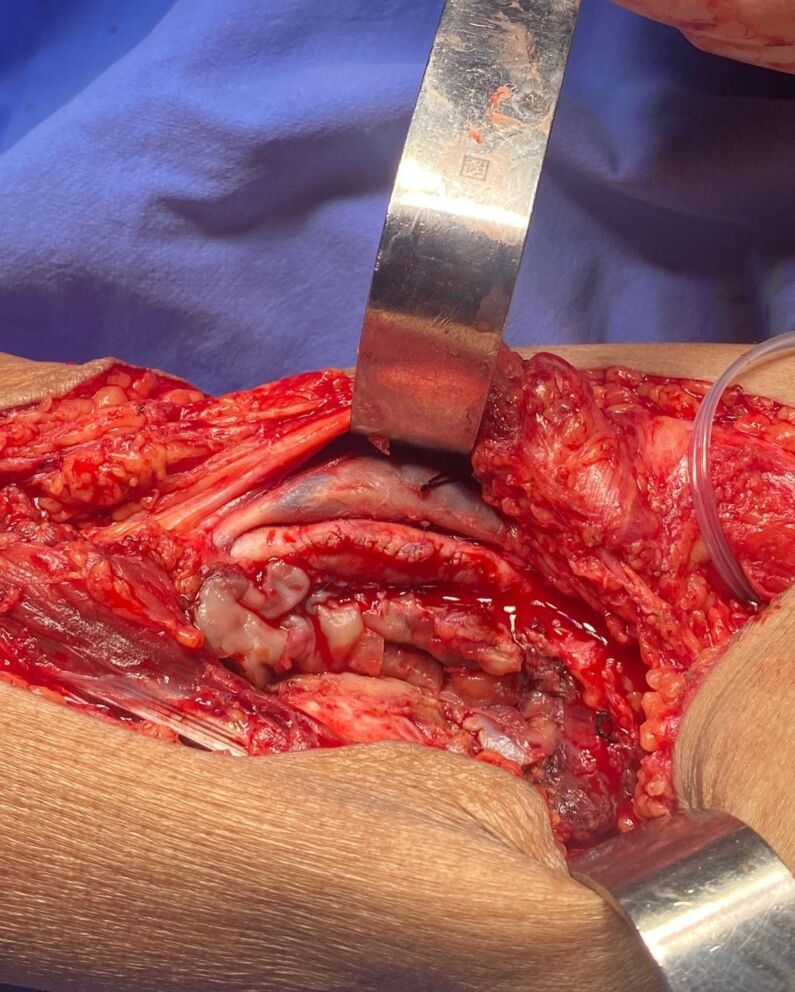
Result after arterioplasty with bovine pericardium.

Patency of the proximal and distal popliteal artery was confirmed and closure was performed with arterioplasty using a bovine pericardial patch (Figure 7).

Post-surgery, the patient’s extremity was warm, with full femoral and popliteal pulses, and the absence of palpable distal pulses was unchanged. She was discharged after four days with manageable pain at the surgical site. At 1-month follow-up, the surgical wound was clean and free from infection, allowing for stitch removal.

**Figure 8:**
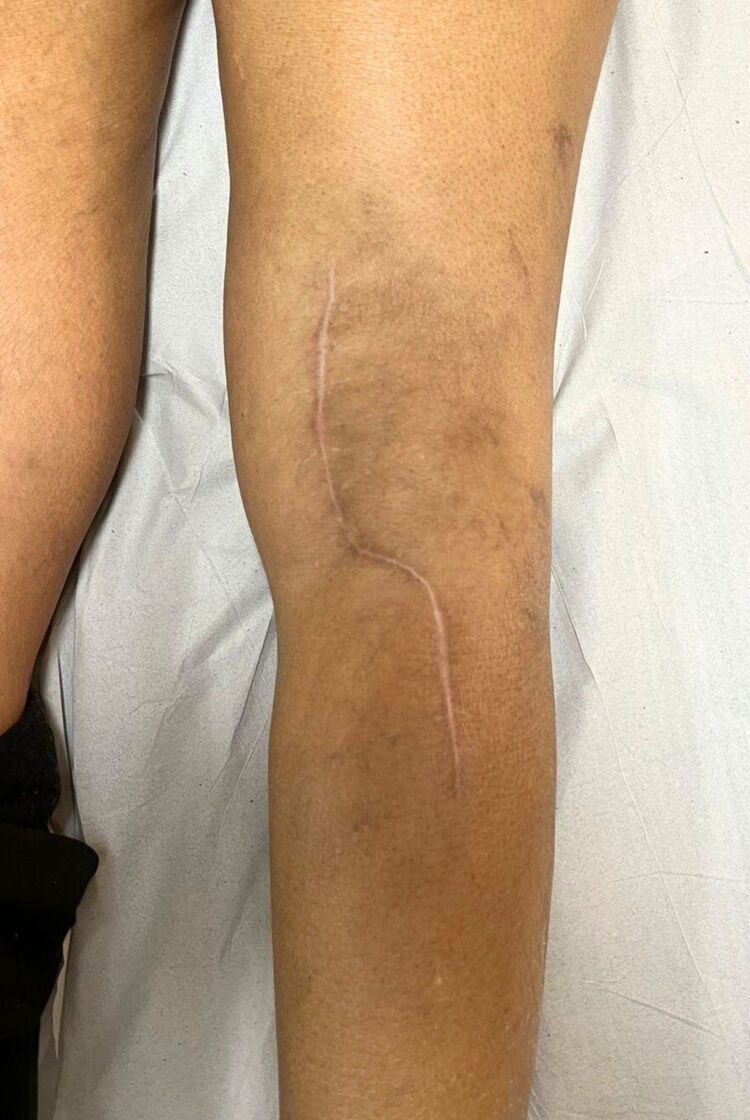
Scar tissue at surgical site at 6-month postoperative follow-up.

**Figure 9:**
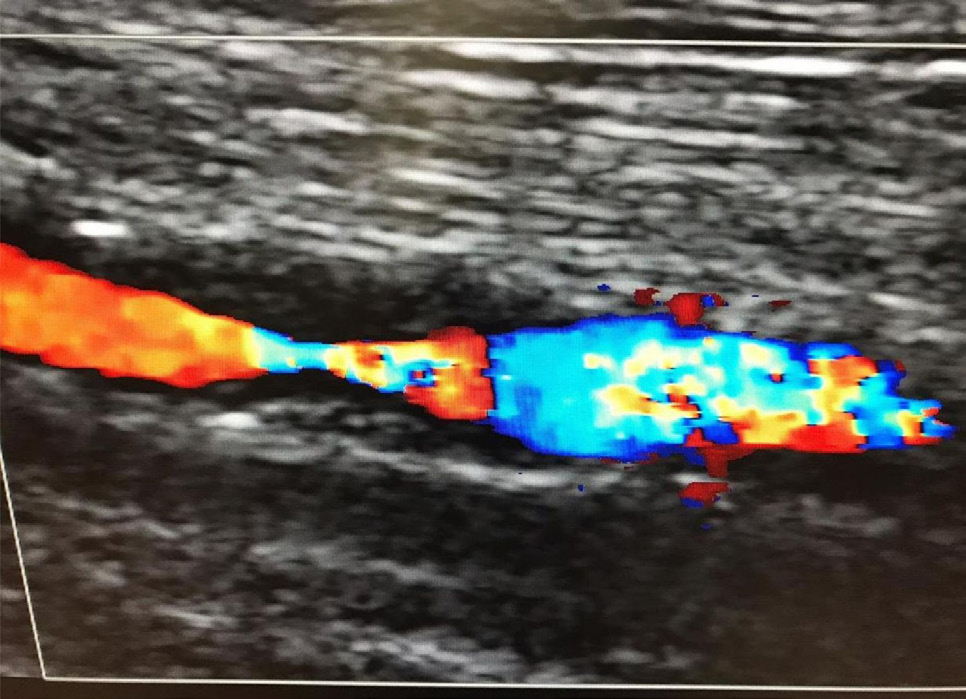
Stenosis at the proximal end of the bovine pericardium patch on color Doppler ultrasound.

**Figure 10:**
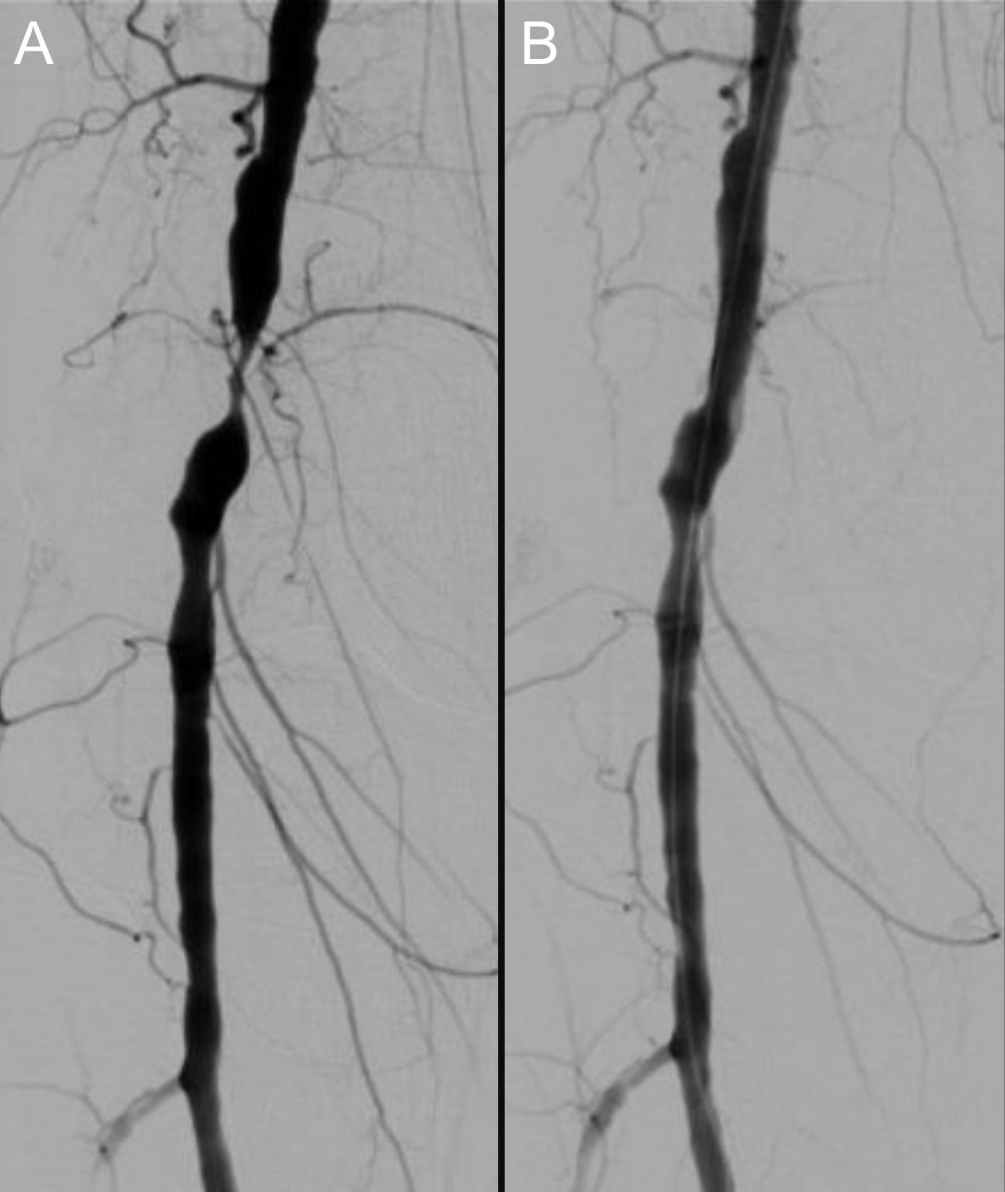
(A) Critical stenosis in the P2 segment of the popliteal artery before and (B) after angioplasty.

Six months post-surgery, the patient’s surgical wound had healed well (Figure 8), although she experienced mild paresthesia in the upper posterior leg. Doppler ultrasound identified stenosis at the proximal end of the bovine pericardium patch. Arteriography confirmed critical stenosis of the popliteal artery’s P2 segment, (Figure 9), which was effectively treated with angioplasty using a 6x40mm balloon (Figure 10).

## DISCUSSION

Popliteal artery pseudoaneurysm is a rare clinical condition and young males constitute the most affected population.^2^ It generally occurs secondary to local trauma,^1^,^2^ orthopedic procedures,^3^,^4^ or bony deformities.^5^ Here, we present the case of an older adult female with a large popliteal artery pseudoaneurysm. Extensive evaluation did not provide significant insights into the etiology of the condition, suggesting an idiopathic pseudoaneurysm.

There are cases in which popliteal artery pseudoaneurysms emerge years after a local trauma,^9^ so it is essential to take a compelling clinical history. Additionally, autoimmune conditions like Behçet’s disease can be associated with pseudoaneurysm development, warranting examinations for urogenital ulcers, eye inflammation, and skin lesions.^10^ In our case, the patient denied any relevant injuries or eye or skin issues and her autoimmune laboratory tests were negative.

The most common symptoms of popliteal artery pseudoaneurysm are local discomfort and the presence of a pulsatile swelling at the popliteal fossa.^2^ While physical examination is often revealing, imaging is crucial for definitive diagnosis because of the potential for atypical presentations.^11^ Arteriography and CT angiography are the most common imaging modalities.^2^ In this case, arteriography was used after color Doppler ultrasound evaluation because it is better able to evaluate distal peripheral artery disease when an interventional procedure is planned. Confirmation of the diagnosis was not pursued with pathology after surgical resection, given the clear preoperative clinical presumption and imaging findings. Upon surgical resection, the aneurysm sac did not show signs of infection that would suggest an infectious aneurysm, although a low-virulence infection could not be completely ruled out.

Regarding management, open surgical repair is considered the gold standard for larger and symptomatic pseudoaneurysms or those located in anatomically favorable positions. Repair involves making an incision over the mass and exposing the affected segment of the popliteal artery, with proximal and distal control of the artery in order to avoid unnecessary bleeding. The surgeon can then perform primary closure if the arterial defect is small or use a vein graft to patch larger defects.^12^ However, this is an invasive procedure that requires a larger incision and may have a longer recovery time. In this particular case, we opted for open surgery due to the pseudoaneurysm’s size and configuration and, most importantly, location, which rendered endovascular treatment unsuitable. Additionally, the patient’s overall health and vascular anatomy favored an open surgical approach, allowing for a more controlled and definitive repair. A bovine pericardium patch was used to close the arterial defect, considering its durability and compatibility with the patient’s vessel size.

Endovascular repair has emerged as a promising minimally invasive option for treating popliteal artery pseudoaneurysms. One of the main limitations is the anatomical complexity of the popliteal artery, which can pose challenges during catheter-based interventions. The vessel’s tortuosity and diameter mismatch could impede precise placement of stent grafts. More importantly, the inherent flexibility of the popliteal artery at the affected location adversely affects the long-term patency of stent grafts, which is particularly problematic in patients with distal peripheral artery disease. Additionally, endovascular repair may be limited by the size and morphology of the pseudoaneurysm. Large or complex pseudoaneurysms may not be amenable to endovascular treatment, requiring open surgical repair due to mass effect. Lastly, the long-term durability and effectiveness of endovascular repair for popliteal artery pseudoaneurysms are still being studied and careful surveillance is necessary to monitor for potential complications and recurrence.^6^,^12^

In summary, popliteal artery pseudoaneurysm, particularly of idiopathic origin, is a rare and clinically challenging condition, with limited reports in the literature. This report highlighted a case of popliteal artery pseudoaneurysm in a 78-year-old female, in which extensive investigation failed to identify any typical etiological factors, thereby classifying it as idiopathic. The successful management of this case with open repair using a bovine pericardium patch demonstrates the effectiveness of open surgery in such atypical presentations. The patient’s postoperative course, which included development and subsequent treatment of proximal patch stenosis, further emphasizes the importance of vigilant follow-up in management of this rare entity.
